# T102. AN INVESTIGATION OF SCHIZOPHRENIA-BIPOLAR SUBGROUPS WITH GENETIC AND PROGNOSTIC VALIDATION

**DOI:** 10.1093/schbul/sby016.378

**Published:** 2018-04-01

**Authors:** Dominic Dwyer, Janos Kalman, Monika Budde, Urs Heilbronner, Anne Ruef, Heike Anderson-Schmidt, Katrin Gade, Nikola Mueller, Ivan Kondofersky, Sergi Papiol, Peter Falkai, Thomas G Schulze, Nikolaos Koutsouleris

**Affiliations:** 1 Ludwig Maximilian University; 2 University Medical Centre Göttingen; 3 Institute of Computational Biology

## Abstract

**Background:**

Separation of individuals into schizophrenia and bipolar diagnoses has long been questioned, with some suggesting that the classification impairs the understanding of etiology, the accuracy of prognoses, and treatment selection. In this study, we employed unbiased statistical techniques to identify subgroups of individuals with chronic illness using a large array of variables commonly evaluated at the bedside. We then validated the resulting groups by investigating age of onset, schizophrenia polygenic risk scores (PRS), and functional outcomes at a 1-year follow-up period. Our hypothesis was that transdiagnostic subgroups would be stratified based on illness onset whereby individuals with earlier onset would have higher genetic risk loading and poorer functional outcomes.

**Methods:**

Participants were selected from a longitudinal, naturalistic, multi-site project (PsyCourse) designed to investigate psychiatric illness course and outcomes. A total of 329 participants (age(SD)=45.7(12.6); 54% female; years of illness duration(SD) = 13.7(10.3)) with a DSM-IV diagnosis of schizophrenia, schizoaffective disorder, or bipolar disorder were assessed from 17 centers at baseline and 1-year follow-up periods. A clinical battery measuring sociodemographic, illness history, symptoms, cognition, and personality questionnaires (199 variables) was used to subgroup individuals. A non-negative factor analytic consensus clustering MATLAB toolbox was created based on previous methodological work in oncology. PRS were generated using widely used strategies, and differences between resulting subgroups were investigated with MANCOVA controlling for ancestry effects. Differences in functional outcomes were investigated with repeated measures ANOVA.

**Results:**

A 4-subgroup solution was robustly defined as the optimal solution using resampling techniques and cluster validity indices. Diagnoses were mixed in two subgroups, but predominantly bipolar or schizophrenia in the other two. All subgroups had equal illness durations (p>0.05), but the age of onset showed a decreasing trend with the earliest age being linked to two subgroups: a mixed bipolar-schizophrenia group with intermediate levels of general functioning and in a schizophrenia group with low levels of functioning (p<0.001). PRS scores were significantly increased in the early-onset, mixed bipolar-schizophrenia subgroup (p=0.007, uncorrected) and in the schizophrenia group (p=0.025, uncorrected). Prognoses differed between the four groups (p=0.003), with the greatest increases in functional outcomes in a late-onset mixed diagnostic subgroup (p=0.006) and in the schizophrenia group (p=0.002).

**Discussion:**

Four subgroups were detected and our hypothesis was supported by a relationship between earlier illness onset and higher schizophrenia genetic risk loading. While one of the subgroups with an earlier onset mostly consisted of individuals with schizophrenia, the other subgroup was diagnostically mixed. Our results tentatively suggest that transdiagnostic clustering may identify subgroups that could be effectively used to understand etiology and prognoses. Future research will investigate the possibility of differential treatment effects in these subgroups.

